# Lymphoma1Read before the Iowa State Veterinary Medical Association.

**Published:** 1898-02

**Authors:** J. L. Miller

**Affiliations:** Ottumwa, Iowa


### LYMPHOMA.[1]

By J. L. Miller, V.S.,
OTTUMWA, IOWA.

A PROFESSIONAL man's duties are so varied and extensive that he has to do much reading and studying in order to keep abreast of his profession, as well as to maintain an ordinary degree of intelligence upon the social, political, and religious problems, that it is scarcely probable he will remember all the important facts in the several branches with which he has to do. Even those of us who are employed in the lecture-room, and go over the same ground year after year, find it necessary to review, and how much more those of us who are engaged in active practice and other divisions of the profession? Hence, it is my purpose to rehearse some facts in regard to this uncommon class of tumors, with a view to helping those who have not had the opportunity of seeing them, that they may become familiar with their structure and appearance, and be able to distinguish them from other growths of similar character.

During the last year, while engaged in meat-inspection, I have had the opportunity of seeing some rare pathologic lesions, among which is this one of lymphoma. There are a great many pathologic conditions of which we frequently read, but seldom have the opportunity of seeing, and how very unfamiliar we are with them!

Lymphoma, lymphadenoma, lympho-sarcoma, are the three names given to this class of tumors; the first indicating a mild and non-malignant form of the disease, and so really considered an hypertrophy of glandular tissue, while the second and third are synonymous terms and are defined by Williams "as a malignant and fatal growth, occurring in all the patients of a veterinary surgeon, as well as in man, arising from no appreciable cause, being sometimes of a very slow, and sometimes of a very rapid growth, differing from other malignant tumors in this one particular, namely, that it seldom invades but one kind of tissue—that of the

[1] Read before the Iowa State Veterinary Medical Association.

glandular system, but as certainly proving fatal as a tumor presenting all the characteristics of the most potent malignancy."

I have always observed these tumors to originate in the lymphatic glands, to which, in some instances, they bear a marked resemblance in anatomical structure, having a medullary and cortical portion enclosed in a capsule which sends trabeculæ into the interior, forming a matrix in which are lodged the new cellular elements. The cortical portion in some instances is well marked, constituting one-half of the entire growth, while more frequently it is less clearly indicated and seems to lose its reddish color and become of uniform color throughout.

Similar changes take place in the medullary substance, which is occasionally a milky-white in the early part of the development, and finally this white tissue is replaced by the gray as the growth progresses; but this is not uniformly so, because we find more frequently that the gray predominates from the commencement.

The construction of these tumors is very much more delicate than many other varieties, and consequently they offer less resistance. In some cases, when cut through, there issues from the cut surfaces a thin, milky fluid. This class of tumors has been observed in the facial, submaxillary, parotid, and cervical regions, also in the axilla and groin.

I have only once seen an exterior development, and in this instance four or five of these tumors, about as many inches apart, appeared on the side of a mule's neck, along the course of the carotid. These were spherical in shape, and varied in diameter from one to four inches.

Lymphomata develop most frequently internally, and have been seen generalized by Dr. Stewart, of Kansas City, as recorded in the last issue of the *Veterinary Review*, and also by Dr. Daniel D. Lee, as stated in the JOURNAL OF COMPARATIVE MEDICINE for January, 1890. The first observed it in a cow, and the latter in a St. Bernard dog. In both of these instances there was gradual and increasing emaciation.

A single instance of the diffused form has been noticed by the writer in a hog, which was accompanied by general emaciation. Lymphoma, however, is more frequently seen involving a single gland, such as the spleen, liver, kidney, mesenteric, or lymphatic gland. In my observations I have found them most frequently in the lymphatic glands of the sublumbar region, where I have seen them originate in a single gland or involve all in that locality.

They vary in shape and size, being sometimes spherical, while others are oblong, or oval. Some of them weigh a few ounces and others several pounds. They never undergo such degenerate changes as softening, ulceration, calcification, or caseation, as tumors of a malignant character usually do. They destroy the function of the gland or glands in which they are located, and cause death in this way, or by exerting pressure on vital parts, or by producing leucocythæmia.

The probability of successfully exterminating them is very limited, even when their location is such as to make them accessible. As the most of these lymphoid growths which have come under the observations of reputable scientists have proven to be malignant, and as it is impossible for many versed in microscopy to discriminate between the benign and malignant, it is incumbent upon those of us who are engaged as sanitarians and meat-inspectors to become as thoroughly acquainted as possible with the different tumors and their distinguishing characteristics. I have obtained and preserved a few specimens, which I send in company with this paper.

As the object of these annual gatherings is to read and discuss subjects of professional interest, and to interchange individual experiences, I send you fraternal greeting, and hope this paper may have some helpful suggestions.

